# Backbone NMR assignments of the essential oxidoreductase tryparedoxin from the human pathogenic parasite *Trypanosoma cruzi*

**DOI:** 10.1007/s12104-025-10244-3

**Published:** 2025-07-28

**Authors:** Eric Schwegler, Ute A. Hellmich

**Affiliations:** 1 Institute of Organic Chemistry and Macromolecular Chemistry, Friedrich Schiller University Jena, 07743 Jena, Germany; 2 Center for Biomolecular Magnetic Resonance (BMRZ), Goethe University Frankfurt, Max von Laue‑Str. 9, 60438 Frankfurt am Main, Germany; 3 Cluster of Excellence Balance of the Microverse, Friedrich Schiller University Jena, 07743 Jena, Germany

**Keywords:** Chagas disease, *Trypanosoma Cruzi*, Tryparedoxin

## Abstract

Over 7 million people worldwide are affected with Chagas disease, a lifelong debilitating and potentially fatal Neglected Tropical Disease caused by the single cell protozoan parasite *Trypanosoma cruzi*. To maintain viability and to reproduce under the harsh conditions within a host organism, pathogens express a variety of protecting enzymes and virulence factors that can serve as potential drug targets. To protect itself from redox stress, *T. cruzi* takes advantage of a unique thiol metabolism. For instance, a cytosolic peroxide clearance cascade is centered on the conserved oxidoreductase Tryparedoxin (Tpx). Tpx efficiently distributes reducing equivalents across the parasitic cell through the promiscuous yet selective binding of numerous up- and downstream clients. However, the exact structure and binding interfaces of this central protein binding hub remain unknown. To study the redox-dependent structural dynamics of *T. cruzi* Tpx, and its interactions with binding partners, we determined the ^1^H, ^13^C, ^15^N-backbone NMR assignments of the enzyme in the reduced and oxidized state. In agreement with earlier NMR studies on Tpx from related protozoans, we report redox-dependent changes in the enzyme’s dithiol active site that could play a crucial role in the recognition of physiological substrates and should be considered in the rational design of small molecule inhibitors.

To whom correspondence should be addressed: U.A. Hellmich, ute.hellmich@uni-jena.de.

## Biological context

The World Health Organization (WHO) lists more parasitic than viral, bacterial or fungal pathogens combined as causative agents for Neglected Tropical Diseases (NTDs) (WHO 2020). Three of these illnesses, namely Sleeping Sickness, Leishmaniasis, and Chagas disease, are caused by infections with eukaryotic, unicellular protozoans, i.e. *Trypanosoma brucei* (*T. brucei*), several *Leishmania* species (*L.* spp.), and *Trypanosoma cruzi* (*T. cruzi*) that parasitize human and animal hosts (WHO 2020).

Infections with *T. cruzi*, the causative agent of Chagas disease, predominantly occur in South and Central America, where over 7 million people live with a chronic infection while another ~ 100 million are considered at risk (WHO 2025). Climate change and the associated spreading of the insect vectors transmitting *T. cruzi* parasites make it likely that these numbers will increase in the near future (Carmona-Castro et al. 2018; Forsyth et al. 2024). For instance, a steady increase in cases in the Southern part of the US has already been reported (Lynn et al. 2020).

Chagas disease can be cured with Nifurtimox and Benznidazole, but only in the acute phase of infection (6–8 weeks) (Echavarría et al. 2021). While the exact mechanism of action remains unclear, both nitroheterocyclic drugs are thought to generate radical species that are harmful to the pathogen (Wilkinson et al. 2008; Hall et al. 2011), but can also cause severe side effects in the human host (Echavarría et al. 2021). In its progressed, chronic stage, Chagas disease can be highly debilitating, and is associated with fatal neurological, digestive and cardiac disorders if left untreated (Echavarría et al. 2021).

Essential enzymes required for *T.cruzi* transmission, survival, and proliferation in the host could constitute alternative drug targets for the development of safer medications that are more effective in the chronic stage of Chagas disease. Among these proteins, members of the pathogen’s enzymatic peroxide clearance cascade, such as the oxidoreductase Tryparedoxin (Tpx), are promising drug targets because they are essential for the parasite’s infectivity and its antioxidative defense against the host’s immune system (Piñeyro et al. 2011; González-Chávez et al. 2019).

While Tpx is highly conserved across trypanosomatids, it diverges from its human counterparts Thioredoxin and Glutaredoxin both structurally and in terms of substrate specificity (Reckenfelderbäumer and Krauth-Siegel 2002). These differences can be used to design inhibitors that selectively interact with the parasitic protein (Fueller et al. 2012). Trypanosomal Tpx features a WCPPC active site motif that undergoes reversible thiol exchange reactions, and was shown to be susceptible to covalent inhibitors (Gommel et al. 1997; Fueller et al. 2012; Wagner et al. 2019; Dietschreit et al. 2020). We and others have previously determined the backbone NMR assignments of Tpx from *T. brucei*, the causative agent of Sleeping Sickness, and *Crithidia fasciculata* (*C. fasciculata*), a non-human parasitizing trypanosomatid (Krumme et al. 2002; Wagner et al. 2017). Furthermore, crystal structures of Tpx from *T. brucei* (e.g. PDB: 1O73, 6GXG) (Alphey et al. 2003; Wagner et al. 2019) and *L. major* (PDB: 3S9F) (Fiorillo et al. 2012), as well as X-ray and NMR structures of *C. fasciculata* Tpx exist (e.g. PDB: 1QK8, 1OKD) (Alphey et al. 1999; Krumme et al. 2003). However, no structure of *T. cruzi* Tpx has been described to date and none of the available Tpx structures accurately represents the reduced form of the protein, which is the dominant redox state in a parasitic cell and has relevance for drug design because it can bind electrophilic molecules (Fueller et al. 2012). As a prerequisite for future functional and drug interaction studies, and to shed light on putative differences between Tpx enzymes from human pathogenic and non-human pathogenic protozoans, we herein report the backbone NMR assignments of *T. cruzi* Tpx in the oxidized and reduced state.

## Methods and experiments

### Protein expression and purification

All chemicals were obtained from Carl Roth unless specified otherwise. The WT Tpx gene from *T. cruzi* (UniProt: Q4D1B8) was synthesized and cloned into a pETtrx_1b vector (GenScript, Piscataway Township, NJ, USA) to heterologously express Tpx as His_6_-tagged Trx (Thioredoxin) fusion protein in *E. coli* BL21Gold(DE3) cells (Agilent, Santa Clara, CA, USA). Transformed bacteria were grown in 1 L LB medium at 37 °C until an OD_600_ of 1.0, centrifuged (5000 rpm, 10 min, RT), and resuspended in 250 mL M9 minimal medium (Marley et al. 2001) supplemented with ^15^NH_4_Cl (1 g/L, Eurisotop) and D-glucose-^13^C_6_ (2 g/L, Cambridge Isotope Laboratories) as the sole nitrogen and carbon sources. Expression of the Trx/Tpx fusion protein was induced with 150 µM IPTG (isopropyl β-D-1-thiogalactopyranoside) and carried out at 20 °C for 20 h. Then, bacteria were harvested via centrifugation (5000 rpm, 10 min, 4 °C), and the cell pellet was flash-frozen with liquid nitrogen and stored at -20 °C.

To purify Tpx, the cell pellet was resuspended in aqueous lysis buffer (50 mM NaPi pH 7.5, 300 mM NaCl), and supplemented with a protease inhibitor cocktail, DNase, RNase, lysozyme and 50 µM PMSF (phenylmethylsulfonyl fluoride) (all Sigma Aldrich). Cells were disrupted with a TT13 sonotrode attached to a Sonoplus SH213G (Bandelin, Berlin, Germany), and cell debris was pelleted via centrifugation (20000 rpm, 45 min, 4 °C). The supernatant containing the Trx/Tpx fusion protein was applied to a gravity flow column with NiNTA (nickel-nitrilotriacetic acid) agarose beads (Qiagen, Hilden, Germany), washed with 10 CV (column volumes) lysis buffer, 10 CV lysis buffer + 10 mM imidazole, and eluted with 4 × 2 CV lysis buffer + 200 mM imidazole. His-tagged TEV protease was added to the Trx/Tpx fusion protein in a molar ratio of 1:40, and the reaction mixture was dialyzed against SEC buffer (25 mM NaPi pH 7.5, 150 mM NaCl) at 4 °C overnight. Tpx was separated from TEV protease and the Trx-His_6_ cleavage product using a second gravity flow Ni-NTA agarose column. The flow-through was combined with washing fractions (3 × 1 CV with SEC buffer + 5 mM imidazole), reduced via the addition of 2 mM DTT (1,4dithiothreitol), concentrated, and applied to a size exclusion column (HiLoad 16/600 Superdex 75 pg (Cytiva, Freiburg, Germany)). ^13^C, ^15^N-labeled Tpx was eluted using SEC buffer, and the purity of collected fractions was checked via SDS-PAGE. Selected fractions were combined, concentrated, flash-frozen in liquid nitrogen, and stored at -20 °C.

### NMR spectroscopy

NMR samples contained 330 µM ^13^C, ^15^N-labeled *T. cruzi* Tpx in SEC buffer (25 mM NaPi pH 7.5, 150 mM NaCl) supplemented with 10% v/v D_2_O (Eurisotop) and 0.15 mM DSS (2,2dimethyl-2-silapentane-5-sulfonic acid). To obtain *T. cruzi* Tpx in the oxidized and reduced state, the protein NMR samples were incubated with either 2-fold molar excess of hydrogen peroxide or 2 mM TCEP (tris(2-carboxyehtyl)phosphine). All NMR spectra were recorded at 298 K with a Bruker Avance Neo 800 MHz spectrometer equipped with a TCI cryoprobe (Bruker Biospin, Ettlingen, Germany). ^1^H, ^15^N-HSQC, HNCA and HN(CO)CA experiments were recorded using the standard pulse sequences, and processed using TopSpin 4.1 (Bruker Biospin, Ettlingen, Germany). Data analysis was carried out in CcpNMR analysis 2.5.2 (Vranken et al. 2005), and the overall redox-induced chemical shift perturbation in the ^1^H-, and ^15^N-dimension (Δδ_HN_) was calculated for each residue using the following equation (Mulder et al. 1999):

$$\Delta\delta_{HN}=\sqrt{\Delta\delta_{H}^{2}+\left(\frac{\Delta\delta_{N}}{6.51}\right)^{2}}$$

where ∆δ_H_ and ∆δ_N_ are the ^1^H- and ^15^N-chemical shift difference, respectively.

Based on the backbone chemical shifts of *T. cruzi* Tpx in the oxidized and reduced state, the probability of each residue to adapt secondary structure (α-helix or β-sheet) was determined with the TALOS-N webserver (Shen and Bax 2013).

### Extent of assignment and data deposition

**Fig. 1:**
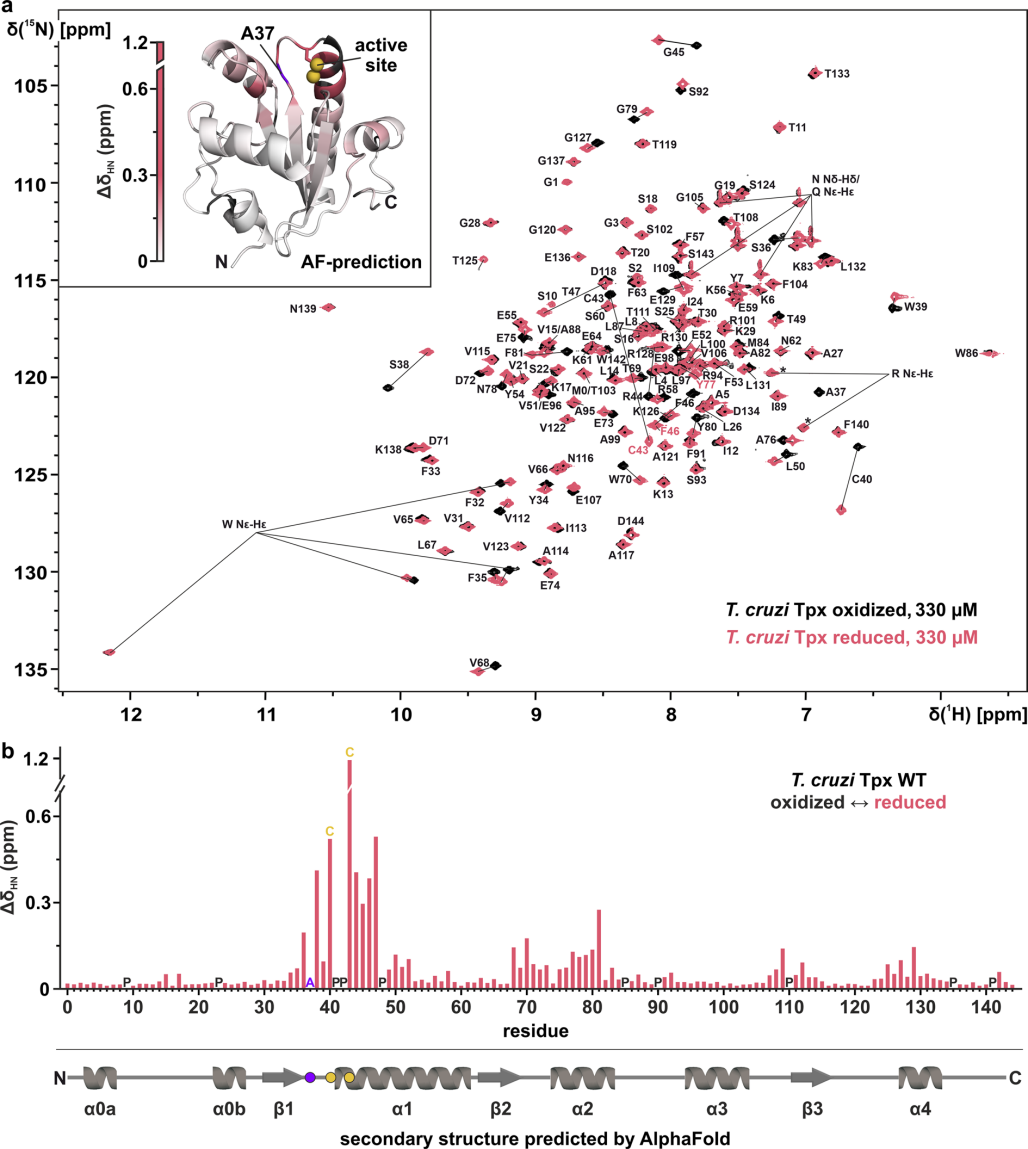
Backbone NMR assignment of *Trypanosoma cruzi* Tpx and redox-induced chemical shift perturbation. **a**, 2D ^1^H, ^15^N-HSQC spectra of ^13^C, ^15^N-labeled *T. cruzi* Tpx in the oxidized (black) and reduced state (red), recorded at 298 K on an 800 MHz spectrometer equipped with a cryogenic triple resonance probe, using 330 µM protein samples. The assignments are given in single letter code following the numbering scheme for *T. cruzi* Tpx according to UniProt (entry Q4D1B8) (The UniProt Consortium 2025) and TriTrypDB (entry TcCL_NonESM13198) (Aslett et al. 2010). Aliased peaks are marked with an asterisk. For the protein sequence, see Fig. 2. Inset: The redox-induced chemical shift perturbation in the ^1^H- and ^15^N-dimension (Δδ_HN_) was mapped onto the AlphaFold model of oxidized *T. cruzi* Tpx (AF-Q4D1B8-F1-model_v4) (Jumper et al. 2021; Varadi et al. 2022). The sulfur atoms of the active site cysteines 40 and 43 are shown as yellow spheres, and the backbone of alanine 37, which could not be assigned in the reduced state, is highlighted in purple. **b**, Redox-induced chemical shift perturbation (Δδ_HN_) for the *T. cruzi* Tpx construct, which includes an N-terminal scar from the cleaved purification tag (see main text for details). Proline residues, the active site cysteines and alanine residue 37 are marked with their respective one letter code in the protein sequence. A topology model following the AlphaFold prediction is shown in the bottom. Circles mark the locations of A37 (purple) and the active site C40 and C43 (yellow)

Due to the high prevalence of Chagas disease worldwide, and the importance of Tpx as the central oxidoreductase of the causative parasite, we report the ^1^H, ^13^C, and ^15^N-backbone resonance assignments of *T. cruzi* Tpx in the oxidized and reduced state (Fig. 1). To obtain the reduced form, ^13^C, ^15^N-labeled *T. cruzi* Tpx was treated with TCEP. To obtain the oxidized state, the protein was incubated with hydrogen peroxide, leading to the formation of a disulfide bond between residues C40 and C43 in the WCPPC active site motif.

In total, 144 (= 98%) of non-proline ^1^H, ^15^N resonances for the reduced and 145 (= 99%) for the oxidized enzyme could be assigned through standard triple resonance pulse sequences (Fig. 1a). For both states, NH resonances from the two N-terminal residues were not visible in the spectrum and, for the reduced state, the NH resonance of alanine residue 37 was also missing. The Cα resonances of 144 (= 98%) *T. cruzi* Tpx residues could be assigned for the reduced, and 145 (= 99%) for the oxidized state. In both cases, Cα resonances for the N-terminal glycine (residue -2) and proline residue 41 were not visible in the 3D spectra and, for the reduced state, the Cα resonance of proline residue 42 was also missing. Indole NH resonances of the tryptophan sidechains were identified based on our previous NMR assignment of the orthologous Tpx from *T. brucei* (Wagner et al. 2017). Arginine sidechain (Nε-Hε) signals appeared as aliased peaks (marked with an asterisk) and were identified by their ^1^H-, ^13^C-, and ^15^N-chemical shifts. Furthermore, the sidechain amide resonances of asparagines and glutamines were identified by recording phase-sensitive ge-2D multiplicity-edited HSQCs using echo-antiecho experiments.

The redox-induced chemical shift perturbations in the ^1^H- and ^15^N-dimension (Δδ_HN_) were mapped onto the tertiary (Fig. 1a, inset) and secondary structure (Fig. 1b) of *T. cruzi* Tpx predicted by AlphaFold (Jumper et al. 2021; Varadi et al. 2022). The largest chemical shift perturbations between the two redox states occurred for residues in the active site and were particularly pronounced for cysteine residues 40 and 43, while smaller chemical shift perturbations were observed for regions that are, according to the AlphaFold prediction, in close vicinity to the active site (e.g. residues 68–81, 107–112, and 125–132). As expected, the per-residue chemical shift perturbations showed that the reduction of the disulfide bond in the Tpx active site affected the chemical environment of the surrounding residues, possibly by the formation of a thiolate anion or a gain in structural flexibility. Interestingly, we observed highly similar redox-induced chemical shift perturbations in our previous assignment of *T. brucei* Tpx (Wagner et al. 2017), suggesting that a redox switch in the dithiol active site has similar implications for both orthologous, parasitic oxidoreductases.

**Fig. 2:**
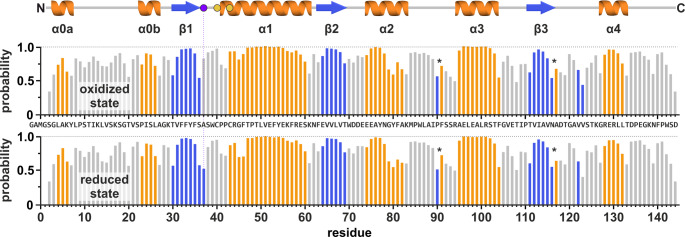
Chemical shift-derived secondary structure prediction of oxidized and reduced *T. cruzi* Tpx compared to the AlphaFold model. Using Talos-N (Shen and Bax 2013), the secondary structure propensity for every residue in *T. cruzi* Tpx (without the N-terminal TEV protease cleavage scar “GAMG”) was determined for the oxidized and reduced state. The highest-ranking secondary structure element for each residue is shown as bar graph (α-helix: orange; β-sheet: blue; loop: grey). For comparison, an AlphaFold-based topology model (Jumper et al. 2021; Varadi et al. 2022) of Tpx starting with native residue 2 is shown on top, with colored circles highlighting the positions of the active site cysteines (yellow) and alanine residue 37 (purple). The AlphaFold and Talos-N secondary structure propensities are in good agreement with each other, with no major changes between redox states. Importantly, the Talos-N prediction for Tpx in the reduced state lacks the ^1^H-, and ^15^N-chemical shifts of A37 which should be considered when comparing the secondary structure probability for this residue in both redox states (indicated with a purple dotted line)

Next, we analyzed whether the *T. cruzi* Tpx secondary structure predicted by AlphaFold for the oxidized state matches the protein structures observed in solution. To this end, we used Talos-N (Shen and Bax 2013) (Fig. 2). The Talos-N secondary structure prediction based on our measured protein backbone chemical shifts (NH and Cα) was very similar for the oxidized and reduced state, and in excellent agreement with the AlphaFold model of the oxidized state (Jumper et al. 2021; Varadi et al. 2022). The β-strand probability of alanine residue 37 seemed slightly increased for the reduced compared to the oxidized state (Fig. 2, vertical dotted line), however, a direct comparison between redox states cannot be made for this residue since A37 did not feature an NH resonance in the ^1^H, ^15^N-HSQC spectrum of the reduced protein. Similarly, in our NMR analysis of the orthologous Tpx from *T. brucei*, we could only detect a strong ^1^H, ^15^N-NMR signal for A37 in the oxidized but not the reduced state (Wagner et al. 2017). Assuming peak broadening as primary cause for the absence of an A37 backbone peak, it is tempting to speculate that this residue becomes more dynamic when the disulfide bond in the active site is reduced.

For two residue pairs (indicated with an asterisk in Fig. 2), TalosN predicted β-sheet probability for the first, and α-helix probability for the second residue. Based on the AlphaFold model of the protein, these two short stretches are part of β-turns featuring ψ and φ dihedral angles that resemble the typical angles expected for secondary structure elements, thereby explaining the prediction outcome. Moreover, Talos-N predicted minor β-strand probability for residue 122 that, according to the AlphaFold prediction, would align in an antiparallel fashion with β-strand 3. Interestingly, in Tpx crystal structures from the related parasites *T. brucei* (Alphey et al. 2003; Wagner et al. 2019), *L. major* (Fiorillo et al. 2012) and *C. fasciculata* (Alphey et al. 1999; Hofmann et al. 2001; Krumme et al. 2003), a fourth β-strand is formed by residues 122–124.

In summary, the backbone assignments of the essential oxidoreductase Tpx from human pathogenic *T. cruzi* parasites not only shed light on the protein structure and consequences of a redox switch in the active site, but also lay the groundwork for detailed studies of drug interactions targeting the causative agent of a debilitating disease.

## Data Availability

The assignments of the wildtype *T. cruzi* Tpx in the oxidized and reduced state have been deposited in the Biological Magnetic Resonance Data Bank (Hoch et al. 2023) (BMRB, https://bmrb.io/) under the accession numbers 53146 and 53147.
